# The impact of oncogenic RAS on redox balance and implications for cancer development

**DOI:** 10.1038/s41419-019-2192-y

**Published:** 2019-12-18

**Authors:** Jonathan K. M. Lim, Gabriel Leprivier

**Affiliations:** Institute for Neuropathology, Medical Faculty, Heinrich Heine University, Moorenstr. 5, 40225 Düsseldorf, Germany

**Keywords:** Oncogenes, Cell signalling

## Abstract

The *RAS* family of proto-oncogenes comprises *HRAS*, *KRAS*, and *NRAS*, which are among the most mutated genes in human cancers. The *RAS* family genes encode small GTPases that coordinate key signaling pathways in response to growth factors. Mutations in *RAS* result in a constitutively active form of the protein that supports cellular transformation and tumorigenesis. The mechanisms of oncogenic RAS-mediated transformation encompass uncontrolled proliferation and inhibition of cell death through overactivation of the RAF-MEK-ERK and the PI3K-AKT pathways, respectively. In addition, the control of redox balance by RAS has also been proposed to play a role in its oncogenic properties. However, the exact role of redox balance in mediating mutant RAS transformation is still under debate. Here, we present, on one hand, the involvement of pro-oxidant components in oncogenic RAS transformation, such as NADPH oxidases and mitochondrial reactive oxygen species, and how these promote transformation. On the other hand, we describe the contribution of antioxidant components to mutant RAS transformation, including Nrf2, glutathione biosynthesis and xCT, as well as the mechanisms by which antioxidant programs drive transformation. Finally, we aim to reconcile the seemingly opposite effects of oncogenic RAS on redox balance and discuss a model for the complementary role of both pro-oxidant and antioxidant pathways in mutant RAS-driven tumor progression.

## Facts

- Superoxide and mitochondrial ROS mediate oncogenic RAS transformation
- Cyclooxygenase-2 supports mutant RAS-induced ROS generation
- Oncogenic RAS reprograms metabolism to favor glutathione biosynthesis and increase NADPH/NADP^+^ ratio
- Induction of the master regulators of antioxidant response NRF2 and xCT promote oncogenic RAS transformation

## Open questions

- How is the control of pro-oxidant and antioxidant pathways by oncogenic RAS coordinated to drive tumorigenesis? Is it sequential and dependent on tumor stages? What is the overall impact on the cellular redox status?
- Do RAS proteins, HRAS, KRAS, and NRAS, regulate distinct redox pathways? Is it tumor-type specific?
- Which redox components can serve as targets for treating RAS-driven human cancers?

## Introduction

A single base substitution in the *HRAS* gene was the first somatic mutation detected in human cancer^1,2^. More than 30 years later, genetic alterations in *RAS* genes, which comprise *HRAS*, *KRAS*, and *NRAS*, are well established as one of the most common oncogenic mutations in cancer, being found in ~30% of all human tumors^3^. In spite of the fact that RAS has been extensively studied, with over 40,000 scientific articles published in the last three decades alone, oncogenic RAS-driven tumors are still widely considered to be difficult-to-treat. Interestingly, while these sobering statistics have been highlighted in the literature countless times, little has changed for patients who are diagnosed with cancers of this genetic subset, who still face dismal prognoses. This underscores the immense difficulty in trying to target RAS and the impetus that is needed to identify novel therapeutic strategies. Therefore, delineating by which mechanisms RAS induces transformation and tumorigenicity is of critical importance.

## RAS effectors and downstream signaling

**Fig. 1:**
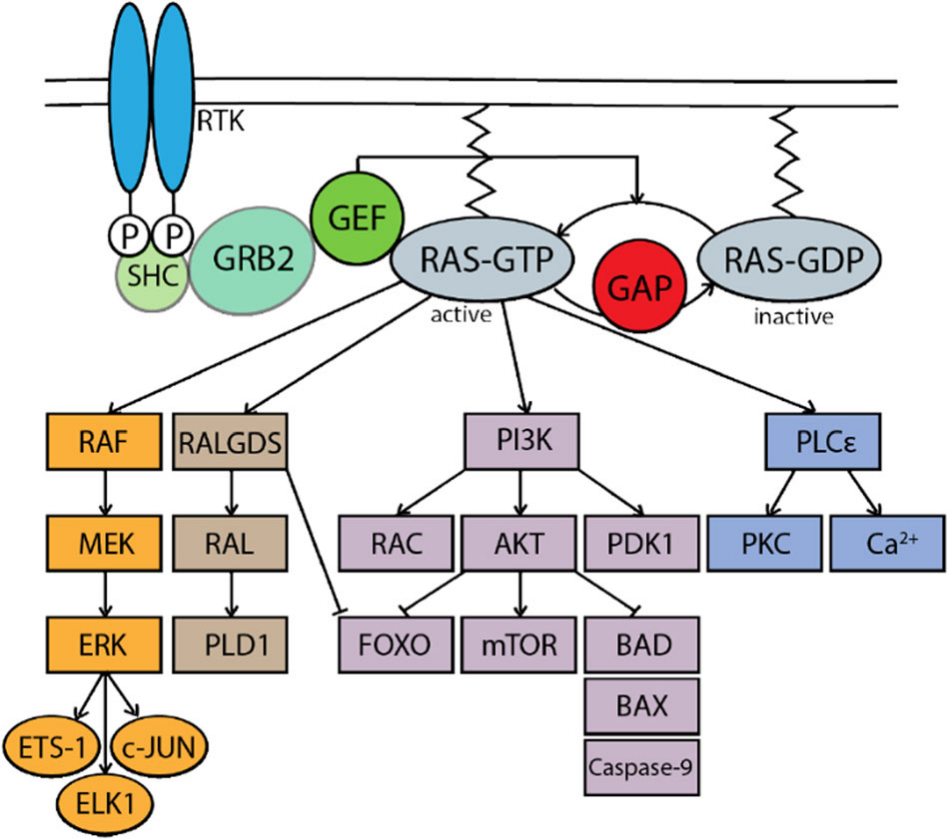
Downstream canonical signaling pathways of oncogenic RAS effectors. RAS signaling is initiated by upstream growth factor receptors and receptors tyrosine kinase (RTK) activation, leading to recruitment of guanine nucleotide exchange factor (GEF) by Src homology 2 domain containing transforming protein (SHC) and growth factor receptor-bound protein 2 (GRB2), which substitutes GDP with GTP to activate RAS. Once in its active state, or in the case of activating mutations, RAS can engage its downstream effectors including but not limited to phosphoinositide 3-kinase (PI3K), rapidly accelerated fibrosarcoma proto-oncogene (RAF), Ral guanine nucleotide dissociation stimulator (RALGDS) and phospholipase C-epsilon (PLCε).

At the cellular level, RAS is a small GTPase protein that is tethered to the plasma membrane via a farnesyl group and relays signals from cell surface receptors to downstream cytosolic effectors constituting several canonical signaling pathways (Fig. 1). The most described of these is the RAF–MEK–ERK pathway, which is perceived as a central regulator of cell cycle progression and proliferation (Fig. 1). Specifically, active RAS–ERK signaling leads to the formation of FOS–JUN heterodimers, which is the process by which the AP-1 transcription factor is activated. Notably, AP-1 is a key regulator of cyclin-D that enables cells to progress through the G1 phase and into the S phase of the cell cycle^4^. Another well-characterized signaling cascade downstream of RAS is the pleiotropic PI3K-AKT pathway, which is a critical determinant of cell survival and growth. Indeed, PI3K-AKT signaling regulates a host of proteins, a number of which are important components of the cell death machinery, including pro-apoptotic family members BAD, BAX, and BIM, caspases, and FAS ligands^5,6^, as well as mTOR, the master regulator of mRNA translation (Fig. 1). Given the involvement of RAF–MEK–ERK and PI3K–AKT in a wide variety of biological processes in addition to cellular survival and proliferation, such as cellular differentiation, migration, and angiogenesis, these signaling pathways are prototypical for other RAS-mediated signal transduction pathways. These include the RALGDS cascade, which controls endosomal trafficking and receptor-mediated endocytosis, and Phospholipase Cε, which is involved in PKC activation and the mobilization of intracellular calcium stores, an integral step in calcium signaling^7^ (Fig. 1).

In cancer cells, the occurrence of mutations in *RAS* results in a conformational change in which the protein is locked into a permanently GTP-loaded state (Fig. 1). This causes constitutive activation of the above-mentioned signaling cascades leading to deregulated proliferation, evasion of apoptosis, and a plethora of other processes that contribute to oncogenic transformation^7^. In recent years, it has become widely appreciated that oncogenic RAS signaling can also influence intracellular redox balance, which can be described as the balance between reactive oxygen species (ROS) and antioxidants, to drive malignant transformation^8,9^. The impact of oncogenic RAS on the different components of the intracellular redox balance and its consequences on tumorigenicity is discussed below.

## ROS and the intracellular redox balance

ROS are chemically reactive, oxygen-containing molecules comprised of free radicals, including superoxide (O_2_^−^), hydroxyl radicals (HO), and nitric oxide (NO), as well as non-radical molecules including hydrogen peroxide (H_2_O_2_), peroxynitrite (ONOO^−^), and hydroxide ion (OH^−^)^10,11^. The fine-tuning of intracellular redox through the balance between ROS-generating and ROS-scavenging programs is critical for cellular homeostasis. At low-to-moderate levels, ROS induces various biological processes, such as proliferation, differentiation, and stress-response activation^12–14^. On the other hand, excessive levels of ROS (leading to oxidative stress) may result in widespread damage to DNA, RNA, proteins, and lipids, ultimately causing senescence, cell death, uncontrolled proliferation, malignant transformation, and metastasis^15,16^. Indeed, a seminal study in 1981 demonstrated that insulin increased tumor cell proliferation by elevating intracellular H_2_O_2_ levels^17^. Further reports showed that ROS can enhance signaling cascades initiated by receptor tyrosine kinases (RTKs) through the reversible inactivation of protein tyrosine phosphatases (PTPs), contributing to the abnormal activation of oncogenic pathways^18^. In addition, ROS promotes widespread genomic instability that may lead to deregulated gene expression or activation of oncogenes, which also supports malignancy^10^. While much of the early literature has supported a role of enhanced ROS levels in the initiation and progression of tumorigenesis, the role of antioxidants in supporting tumor development has only more recently emerged^9,19–22^.

The impact of RAS on redox homeostasis and its contribution to transformation and tumorigenesis are still a matter of debate. A large number of studies have demonstrated that forced expression of RAS leads to an increase in ROS production. This accumulation in ROS was understood to play a dual role, both in the establishment of oncogene-induced senescence, but also as an essential mediator of RAS-induced transformation and tumorigenicity^23,24^. More recently, the scientific literature also supports the notion that oncogenic RAS drives antioxidant programs that drive tumorigenesis. Taken together, it is still unclear how exactly RAS modulates redox balance, or whether it is predominantly pro-oxidants or antioxidants that contribute to RAS transformation. In addition, the precise mechanisms by which the intracellular redox environment influences malignant transformation and tumorigenesis are largely unknown. Here, we discuss the landscape of pro-oxidant and antioxidant programs reported to be controlled by RAS, and attempt to reconcile the seemingly contradictory effects of RAS on the intracellular redox environment and the subsequent impact on tumorigenesis.

## Oncogenic RAS drives pro-oxidant programs to support tumorigenesis

### NADPH oxidase complex

Much of the early literature investigating the impact of oncogenic RAS on the intracellular redox environment has suggested a role for elevated ROS levels as a driver of transformation and tumorigenesis. Irani et al.^25^ first showed that ectopic expression of *HRAS*^*V12*^ in NIH3T3 fibroblasts leads to the production of large amounts of superoxide. They demonstrated that this is correlated with the progression of cells through the cell cycle in a Rac1-dependent manner. Further investigation determined that oncogenic RAS increases superoxide production by upregulating *Nox1* transcription through the MAPK pathway^26^. NOX1 or NADPH oxidase 1 is a member of the NADPH oxidase enzyme family, which is responsible for the catalytic one-electron transfer of oxygen to generate superoxide at the plasma membrane. Indeed, the authors found that suppression of *Nox1* expression abrogates superoxide generation and prevents oncogenic RAS-transformed phenotypes, including anchorage-independent growth and morphological changes, while antioxidant treatments also strongly suppress RAS-induced tumor formation in vivo^26^. These data suggest that NOX1-mediated ROS production is necessary to support RAS transformation and tumorigenesis.

**Fig. 2:**
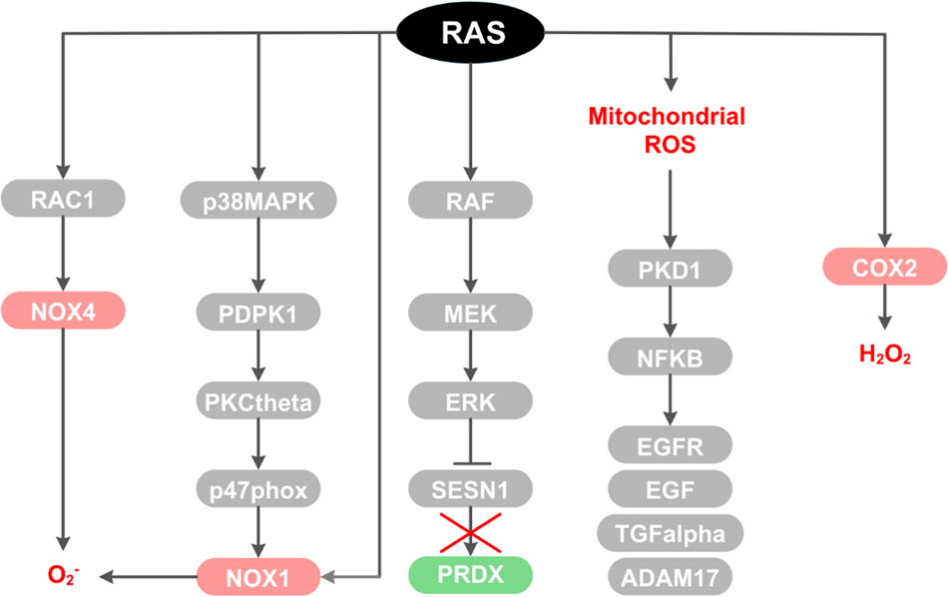
Signaling pathways and mechanisms driving oncogenic RAS induction of cellular pro-oxidant programs. Oncogenic RAS drives multiple pro-oxidant programs ranging from activation of subunits of the NADPH oxidase complex (NOX1/4), inactivation of antioxidants such as sestrin 1 (SESN1), or promoting ROS production from the mitochondria or from cyclooxygenase-2 (COX2).

Alternatively, it was proposed that RAS signaling can directly mediate Nox1 activation independently of stimulating *Nox1* transcription^27^. Specifically, it was demonstrated that *KRAS*^*V12*^ activates p38 Mapk to induce 3-phosphoinositide-dependent protein kinase 1 (Pdpk1), in turn activating protein kinase C δ (Pkcδ). This latter kinase catalyzes the phosphorylation and activation of the p47^phox^ Nox1 subunit, inducing its translocation to the plasma membrane, resulting in Nox1-mediated ROS generation (Fig. 2). More importantly, they showed that inhibition of either p38, Pdpk1, Pkcδ, p47^phox^, or Nox1 suppresses KRAS-induced ROS generation and cellular transformation, as displayed by soft agar colony-formation and tumor-formation assays^27^. Thus, this study demonstrates through a distinct posttranslational mechanism, that KRAS activates NOX1-dependent ROS production, which is necessary to support KRAS-induced cellular transformation.

Similarly, a recent study established that oncogenic RAS-induced ROS formation is dependent on RAC1 and NOX4, a homolog of NOX1, as demonstrated in normal human fibroblasts and in a *HRAS*^*V12*^ transgenic zebrafish model^24^ (Fig. 2). This study further demonstrated that pharmacological inhibition of NOX4 prevents *HRAS*^*V12*^-mediated hyperproliferation and DNA-damage response activation. Similar effects were observed by scavenging ROS generation with N-acetyl cysteine (NAC), arguing that the role of NOX4 in supporting oncogenic RAS transformation is directly related to its ROS-producing function. In addition, Nox4 levels were increased during pancreatic cancer progression in a *KRAS*^*G12D*^-driven mouse model of pancreatic cancer, highlighting a link between high NOX4 expression and advanced stage of a KRAS-driven tumor type^24^.

### Cyclooxygenases

In contrast to these studies, Maciag et al.^28^ found that stable expression of oncogenic KRAS^V12^ in mouse peripheral lung epithelial cells (E10 cells) increases levels of intracellular ROS while superoxide levels remain unchanged, suggesting an alternate means of ROS production that can support RAS transformation. It was postulated that KRAS regulates cyclooxygenase-2 (Cox-2), an enzyme whose activity releases prostaglandin-E_2_ and produces H_2_O_2_ as a by-product (Fig. 2). Indeed, the authors found that Cox-2 protein expression and activity is significantly elevated in mutant *KRAS*^*V12*^ mouse lung epithelial cells and that treatment with a Cox-2 inhibitor results in a concentration-dependent reduction in ROS^28^. More importantly, they observed that *KRAS*^*V12*^-induced ROS generation leads to a significant increase in DNA single-strand breaks in a Cox-2-dependent manner^28^. This is of relevance at advanced stages of cancer, where DNA damage can be accompanied by elevated levels of ROS, due in part to a vicious cycle in which ROS promotes DNA damage and genetic mutations due to defective DNA repair, which leads to further redox imbalances, finally resulting in more aggressive malignant behavior^29^. It is tempting to speculate the reasons for which the above-mentioned studies showed conflicting results regarding the ability of oncogenic RAS to increase superoxide levels^24–26,28^. On one hand, this could be attributed to cell line-specific effects (in previous papers mutant RAS is expressed in mouse and human fibroblasts^24–26^, while Maciag et al. utilized lung epithelial cells^28^). On the other hand, it is also possible that in parallel to the elevation of superoxide levels, oncogenic RAS may also be activating superoxide dismutases (SODs)^30^, which rapidly converts superoxide to H_2_O_2_, thereby causing superoxide levels to appear unchanged. This speculation thus highlights an area for further investigation.

### Repression of antioxidants

Interestingly, besides the direct activation or induction of ROS-producing enzymes, such as NADPH oxidase complexes and COX-2, RAS oncogenic signaling can also promote a pro-oxidant environment by repressing antioxidant molecules. A study, for example, showed that *Nras*^*D13*^-induced ROS upregulation is accompanied by transcriptional repression of the Sestrin gene family^31^, while overexpression of sestrins interferes with ROS induction (Fig. 2). This is in line with the role of sestrins (SESN1, 2, and 3) in the regeneration of cytosolic peroxiredoxins, the enzymatic antioxidants involved in the decomposition of endogenously produced H_2_O_2_. Functionally, the resultant increase in intracellular ROS was shown to cause chromosome instability, as evidenced by an increase in DNA oxidation and the number of chromosome breaks, which may contribute to oncogenic RAS transformation^32^.

### Mitochondrial metabolism and ROS

More recent focus on RAS has shifted toward RAS regulation of mitochondrial metabolism. Several lines of evidence suggest that oncogenic KRAS transformation alters mitochondrial metabolism to increase ROS generation. For instance, Weinberg et al.^8^ showed that the major site of KRAS-induced ROS generation is the Q_o_ site of the mitochondrial complex III and that this mitochondria-derived ROS is critical for oncogenic KRAS-driven cell proliferation and anchorage-independent growth via ERK signaling, independently of oxidative phosphorylation. Furthermore, Liou et al.^33^ elucidated a signaling pathway linking Kras-induced mitochondrial ROS generation to the formation of pancreatic precancerous lesions. Their data showed that *Kras*^*G12D*^-induced mitochondrial ROS leads to the activation of protein kinase D1 (Pkd1) and subsequently NF-κB in mouse primary pancreatic acinar cells. This leads to the induction of epidermal growth factor receptor, *Egfr*, and its ligands *Egf* and *Tgf-alpha*, as well as their sheddase *Adam17*^33^ (Fig. 2). Altogether, this causes the autocrine activation of Egfr signaling, which drives the de-differentiation of acinar cells to a duct-like progenitor phenotype and progression to pancreatic precancerous lesions, known as pancreatic intraepithelial neoplasias (PanINs)^33^. Taken together, these studies provide several insights into how oncogenic RAS drives intracellular pro-oxidant programs to modulate essential molecular pathways, including increased cell proliferation, de-differentiation of cells, genetic instability, and other features of the transformed phenotype to support RAS-mediated oncogenesis (Fig. 4).

## Oncogenic RAS drives antioxidant programs to support tumorigenesis

### Antioxidant enzymes

Given the many studies that suggest a role of RAS in activating pro-oxidant programs to drive tumorigenesis, coupled with epidemiologic studies pointing to an association between dietary antioxidants and a decreased risk for developing cancer^34,35^, cellular antioxidant programs have until recently been unappreciated as mediators of oncogenesis, and to the contrary have been generally considered to have tumor-suppressive function.

**Fig. 3:**
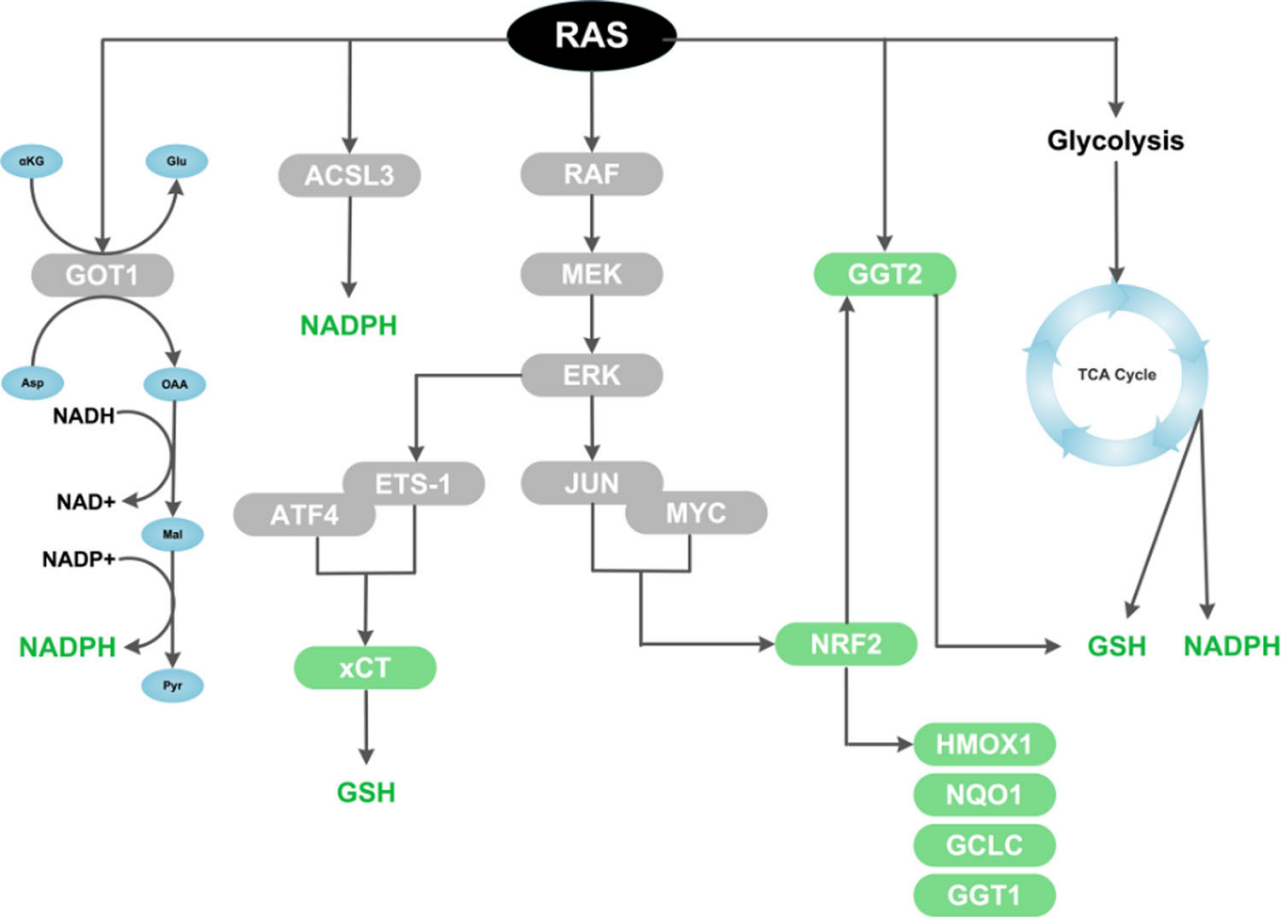
Signaling pathways and mechanisms driving oncogenic RAS induction of cellular antioxidant programs. Oncogenic RAS drives multiple antioxidant programs by altering intracellular metabolism, such as by driving GSH and NADPH production via the TCA cycle, by generating NAPDH through an alternative glutamine metabolic pathway mediated by aspartate aminotransferase (GOT1), or potentially by generating NADPH via a fatty acid oxidation pathway mediated by acyl-coenzyme A (CoA) synthetase long-chain family member 3 (ACSL3). In addition, oncogenic RAS upregulates several key antioxidant proteins, including the light-chain subunit of the system x_c_^−^ transporter (xCT), nuclear factor, erythroid 2-like 2 (NRF2), and gamma-glutamyltransferase 2 (GGT2).

Using functional proteomic approaches, several initial studies provided evidence that KRAS-transformed cells display an upregulation of major antioxidant enzymes, including peroxiredoxin 3, thioredoxin peroxidase, and catalase, which correlated with increased intracellular reduced glutathione (GSH), as well as enhanced detoxification capacity and resistance to apoptosis in response to H_2_O_2_ or formaldehyde^36,37^. Another study showed that KRAS-mediated transformation in prostate epithelial cells upregulates gamma-glutamyltransferase 2 (GGT2), an enzyme involved in the maintenance of glutathione homeostasis^38^ (Fig. 3). KRAS-mediated GGT2 activation also confers resistance to H_2_O_2_-induced apoptosis, and GGT2 expression is dependent on the ERK pathway. These studies laid the foundation for a landmark study by DeNicola et al.^9^ which demonstrated that mutant *Kras*^*G12D*^ expression from an endogenous locus leads to an increase of the antioxidant capacity (indicated by an increase in the ratio of reduced to oxidized glutathione [GSH/GSSG]), and is linked to reduced intracellular ROS levels. In contrast, *Kras*^*G12D*^ expressed from an ectopic promoter reduces the GSH/GSSG ratio and increases ROS, in line with previous studies (as discussed in the previous section). As described in their findings, this discrepancy could be potentially explained by the ability of ectopic *Kras*^*G12D*^, but not of endogenous *Kras*^*G12D*^, to induce *Nox* transcription and therefore NOX complex activity. This finding questions previous studies showing that oncogenic KRAS promotes ROS induction, in which transformation was typically modeled with ectopic expression of mutant *Kras*. It also brings into question whether expression of mutant *Kras* will cause an overall increase or decrease in intracellular ROS levels. The mechanism supporting endogenous *Kras*^*G12D*^ reduction of ROS relies on the control of the transcription factor nuclear factor, erythroid 2-like 2, or Nrf2, widely regarded as the master regulator of antioxidant response^9^. Specifically, the authors showed that endogenous *Kras*^*G12D*^, via the Raf-Mek-Erk-Jun pathway, transcriptionally activates Nrf2 and by extension Nrf2 target genes (*Hmox1, Nqo1, Gclc*, and *Ggt1*) in cells, genetically engineered mouse models (GEMMs) of pancreatic and lung cancer, and in human pancreatic cancer^9^ (Fig. 3). More importantly, it was shown in vivo that Nrf2 deficiency reverses the reduction in ROS due to *Kras*^*G12D*^ and causes a significant reduction in tumor volume and tumor cell proliferation in oncogenic *Kras* mouse models of pancreatic and lung cancer^9^. This highlights the essential role of Nrf2 in driving a robust antioxidant transcriptional program necessary for *Kras*^*G12D*^-initiated tumorigenesis and proliferation. In relation to this, it was also shown that in non-small-cell lung cancer, oncogenic KRAS alters asparagine biosynthesis via the oxidative stress-responsive NRF2 and ATF4 transcription factors, to suppress apoptosis in response to glutamine deprivation and to sustain tumor growth^39^. This implies that in response to nutrient stress, oncogenic KRAS could likewise activate downstream NRF2 and ATF4-dependent antioxidant mechanisms to support tumor progression^39^.

### Non-enzymatic antioxidants

It is worth noting that ROS detoxification by enzymatic antioxidants is a biochemical process that consumes GSH and ultimately NADPH, given that NADPH is required to reduce GSSG and is thus the predominant source of reducing power. Consequently, generation and maintenance of intracellular GSH and NADPH pools is vital for redox homeostasis and potentially for oncogenesis^40^. Indeed, a recent study revealed that oncogenic KRAS promotes a constant supply of NADPH by reprogramming glutamine metabolism via the transcriptional upregulation of aspartate transaminase (GOT1)^41^ (Fig. 3). The authors showed that pancreatic ductal adenocarcinoma (PDAC) cells and tumors are critically dependent on a noncanonical, GOT1-mediated metabolic pathway of glutamine that leads to the cytosolic conversion of glutamine-derived aspartate into oxaloacetate (OAA), malate, and then pyruvate^41^. This pathway increases the NADPH/NADP^+^ ratio and thereby maintains redox balance to sustain PDAC tumor growth^41^. In line with this, another study showed that in mouse embryonic fibroblasts and lung cancer cell lines, as well as in advanced lung tumors, mutant *Kras*^*G12D*^ allelic copy gain (*Kras*^*G12D/G12D*^) leads to a reprogramming of glucose metabolism^42^. This is marked by increased channeling of glucose-derived metabolites into the tricarboxylic acid (TCA) cycle and GSH biosynthesis, leading to enhanced NADPH and GSH levels and ultimately ROS detoxification^42^ (Fig. 3). The *Kras*^*G12D*^ copy gain and associated upregulation of antioxidant capacity was also shown to drive malignant progression and metastatic potential in lung cancer cells and lung tumors in vivo, as the rate of tumor cell proliferation was reduced by treatment in vivo with a glutamate cysteine ligase (GCL) inhibitor (1-buthionine-S,R-sulfoximine (BSO)), which blocks GSH biosynthesis^42^.

Notably, our recent findings demonstrated that oncogenic KRAS expression in mouse fibroblasts confers protection against oxidative stress by enhancing intracellular GSH levels^43^. We reported that this is due to transcriptional induction of *xCT* (*Slc7a11)*, which encodes the xCT light-chain subunit of the system x_c_^−^ transporter, the main cystine transporter involved in providing cystine intermediates for de novo synthesis of GSH (Fig. 3). Mechanistically, we found that the ETS-1 transcription factor downstream of RAS–ERK signaling directly transactivates the *xCT* promoter in synergy with the oxidative stress-responsive ATF4 transcription factor^43^ (Fig. 3). Furthermore, *xCT* knockdown significantly ablated the growth of colonies in soft agar, as well as the growth of tumor xenografts established from KRAS-transformed cells, which correlated with increased oxidative stress and decreased levels of intracellular GSH^43^. Overall, our findings and those of other groups illustrate that oncogenic RAS transformation is supported by downstream induction of antioxidants programs and fine-tuning of the redox balance.

### Metabolic processes

**Fig. 4:**
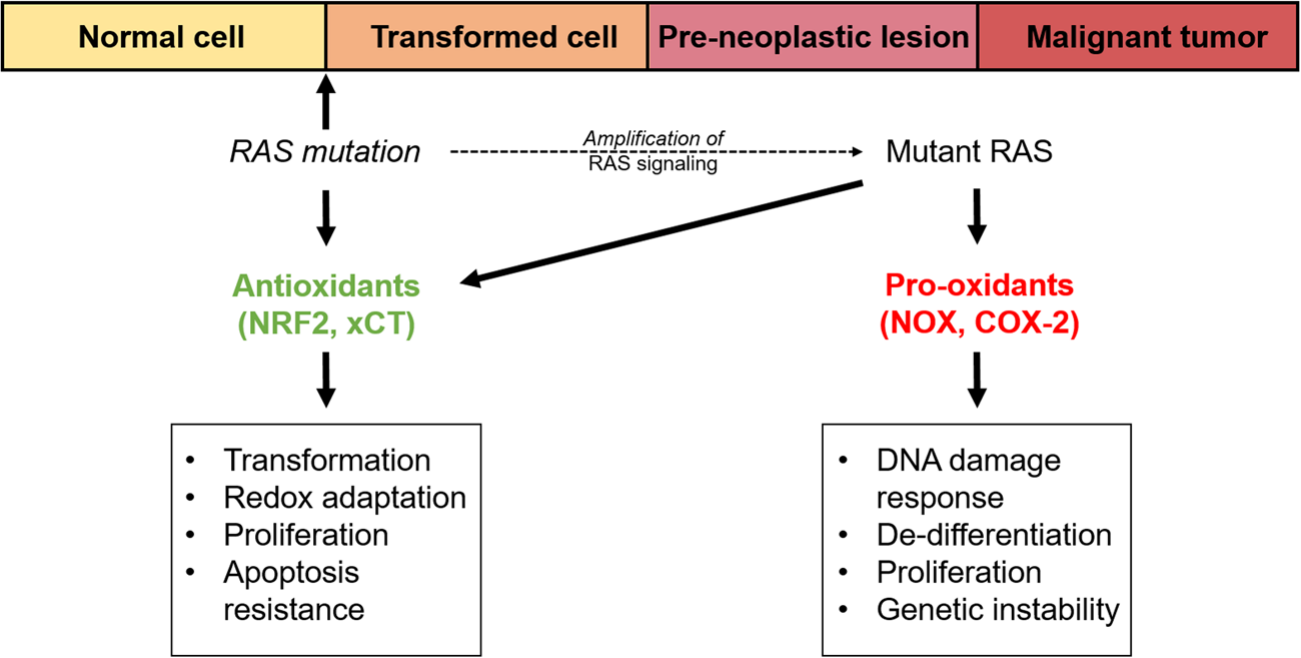
Proposed model for the role of cellular redox homeostasis in oncogenic RAS-mediated tumor initiation and progression. Oncogenic RAS activates antioxidant programs at tumor initiation, leading to redox adaptation, proliferation, and transformation, as well as apoptosis resistance. During tumor progression, oncogenic RAS additionally promotes pro-oxidant programs, which drive DNA-damage response activation, de-differentiation, genetic instability, and proliferation.

The capacity of oncogenic KRAS to rewire metabolic networks is not only limited to glycolytic or glutamine pathways but also lipid biosynthetic processes. Recently, a group reported that mutant KRAS promotes the cellular uptake, accumulation, and beta-oxidation of fatty acids in lung cancer cells, as well as lung tumors, through the upregulation of Acyl-coenzyme A (CoA) synthetase long-chain family member 3 (ACSL3)^44^ (Fig. 3). This has relevance for antioxidant production, as fatty acid oxidation generates acetyl CoA, which is metabolized to produce NADPH^45^, especially under conditions of glucose scarcity. Therefore, it is possible that oncogenic KRAS-driven fatty acid oxidation could support NADPH generation and contribute to intracellular antioxidant capacity, although this mode of NADPH maintenance remains to be defined. These diverse studies highlight the role of oncogenic RAS in enhancing intracellular antioxidant capacity, and support the notion that building antioxidant capacity is in fact critical for oncogenic RAS-mediated tumorigenicity. In addition, evidence suggests that induction of these molecular pathways serve not only as an adaptation to oxidative stress, but may occurs intrinsically to support apoptotic resistance, proliferation, and cellular transformation (Fig. 4).

## Concluding remarks and future perspective

Our understanding of how oncogenic RAS regulates the balance between pro-oxidant and antioxidant programs to support transformation and tumorigenicity has greatly increased over the last two decades. Nonetheless, further investigation is warranted to reconcile their seemingly contradictory effects and to further elucidate the complex interplay of these processes. It is tempting to discount earlier studies which mainly relied on ectopic overexpression approaches to illustrate that oncogenic KRAS promotes pro-oxidant programs (such as NOX), on the basis that they may not accurately recapitulate tumor physiology, and that those findings are now being supplanted by the less artificial endogenous expression models. However, this explanation might be an oversimplification. Whether oncogenic RAS will increase or decrease intracellular ROS is likely to be dependent on multiple factors. In this area, the regulation of RAS on downstream effector signaling in general may shed light on this conundrum. Indeed, even though oncogenic RAS can activate more than 20 different downstream effectors, it has been observed that in any given cell type only a subset of these will be selectively activated, resulting in distinct physiological consequences^46^. Firstly and perhaps not surprisingly, this can be attributed to isoform-specific differences of RAS proteins. Namely, each RAS isoform may be differentially coupled to distinct downstream effectors. For instance, Kras has been demonstrated to activate Raf-1 more efficiently, whereas Hras and Nras are more potent activators of PI3K^47^. Second, it is now widely understood that apart from constitutive activation due to mutation, the expression level of oncogenic RAS can also define phenotypic outcome^48^. Indeed, in addition to the type of expression system employed, whether ectopic or endogenous^49^, the presence of contributing stimuli from the microenvironment can also generate significantly different signaling outputs downstream of RAS. Several studies, for example, have shown that endogenous expression of mutant KRAS alone in GEMMs fails to show any discernible pathological effect, unless coupled with chronic pancreatitis or the presence of inflammatory stimuli, which are necessary to amplify RAS signaling to an effective level^50,51^. Other potential factors that influence downstream RAS effector signaling include cell-lineage dependency^52^, the presence of a wild-type RAS allele^53^, and the interaction of RAS with particular effectors in specific microdomains^49^.

In this light, it can likewise be rationalized that oncogenic RAS is able to regulate a combination of both pro-oxidant and antioxidant programs depending on the context, in order to promote transformation and tumorigenesis (Fig. 4). The induction of these programs may occur in a sequential manner throughout the stages of oncogenesis, with specific redox programs occurring very early during the transformation process, such as in pre-neoplastic tissue, and others occurring at later stages of tumorigenesis. For instance, we speculate a possible scenario of cancer initiation, in which early genetic alterations in KRAS activate cellular processes that drive cellular transformation along with the intrinsic activation of antioxidant mechanisms, such as Nrf2 or xCT^9,43^. Later, as oncogenic RAS signaling is further amplified due to inflammatory stimuli or other stress conditions, oncogenic RAS further activates downstream pro-oxidant pathways such as NOX and COX-2, resulting in the accumulation of mutational events, further increasing genetic instability, de-differentiation, and hyperproliferation, all of which are necessary for tumor progression (Fig. 4). In this increasingly stressful microenvironment, cells that already have an established strong antioxidant response will then be refractory to senescence and cell death, and therefore be able to support continued neoplastic growth^9,21,54^. It is even conceivable that both pro- and antioxidant programs occur in parallel while being confined to separate compartments or microdomains in the cell. In the scenarios put forth above, the absence of either of these antioxidant or pro-oxidant programs will therefore be deleterious for tumor initiation and progression. In support of this notion, Liou et al.^33^ found that even though Nrf2 expression was increased in acinar cells and PanIN lesions driven by oncogenic KRAS, these pre-neoplastic structures still showed significant oxidative damage as indicated by 4-hydroxynonenal staining. So, even though the activation of Nrf2 by oncogenic RAS signaling was present, this did not completely mitigate ROS levels (complete suppression of ROS may not be permissive for proliferation, due to its normal role in cellular signaling), but instead drove antioxidant responses to an extent that was sufficient to prevent cellular senescence or apoptosis, while allowing a threshold of intracellular ROS from pro-oxidant programs to continue exerting other pro-tumorigenic effects.

Overall, the regulation of redox homeostasis by oncogenic RAS to support transformation and tumorigenesis is complex and warrants further study. Nonetheless, the information in this review and our attempt to reconcile the seemingly contradictory effects of pro-oxidant and antioxidant pathways in the context of RAS-induced tumorigenesis should help further our understanding of how mutant RAS modulates a delicate balance between pro-oxidant and antioxidant signals. Ultimately, we expect that the continual investigation in this area will uncover redox-specific vulnerabilities that can help inform novel therapeutic strategies for the treatment of oncogenic RAS-driven cancers.
